# A Novel PiRNA Enhances CA19-9 Sensitivity for Pancreatic Cancer Identification by Liquid Biopsy

**DOI:** 10.3390/jcm11247310

**Published:** 2022-12-09

**Authors:** Weiyao Li, Miguel Gonzalez-Gonzalez, Lara Sanz-Criado, Nuria Garcia-Carbonero, Angel Celdran, Pedro Villarejo-Campos, Pablo Minguez, Roberto Pazo-Cid, Custodia Garcia-Jimenez, Alberto Orta-Ruiz, Jesus Garcia-Foncillas, Javier Martinez-Useros

**Affiliations:** 1 Department of Gastrointestinal Surgery, The Sixth Affiliated Hospital, Sun Yat-sen University, Guangzhou 510085, China; 2 Microbiology Laboratory, Clinico San Carlos University Hospital, 28040 Madrid, Spain; 3 Translational Oncology Division, OncoHealth Institute, Health Research Institute Fundacion Jimenez Diaz, Fundacion Jimenez Diaz University Hospital, Universidad Autonoma de Madrid (IIS-FJD, UAM), 28040 Madrid, Spain; 4 General and Hepatobiliary Surgery Unit, Fundacion Jimenez Diaz University Hospital, 28040 Madrid, Spain; 5 Genetics and Genomics Department, IIS-Fundacion Jimenez Diaz, Universidad Autonoma de Madrid (IIS-FJD, UAM), Center for Biomedical Network Research on Rare Diseases (CIBERER), ISCIII, Bioinformatics Unit, IIS-FJD, UAM, 28040 Madrid, Spain; 6 Department of Medical Oncology, Hospital Universitario Miguel Servet, 50009 Zaragoza, Spain; 7 Area of Physiology, Department of Basic Health Sciences, Faculty of Health Sciences, Rey Juan Carlos University, 28922 Madrid, Spain; 8 Department of Medical Oncology, Hospital Clínico San Carlos, Instituto de Investigación Sanitaria del Hospital Clínico San Carlos (IdISSC), CIBERONC, 28040 Madrid, Spain; 9 Department of Medical Oncology, MD Anderson Cancer Center Madrid, 28033 Madrid, Spain; 10 Faculty of Experimental Sciences, Universidad Francisco de Vitoria, 28223 Pozuelo de Alarcón, Spain

**Keywords:** PIWI proteins, small non-coding RNA, piRNA, PIWIL3, PIWIL4, pancreatic cancer, liquid biopsy, CA19-9, MAPK pathway

## Abstract

Pancreatic cancer is one of the deadliest tumours worldwide, and its poor prognosis is due to an inability to detect the disease at the early stages, thereby creating an urgent need to develop non-invasive biomarkers. P-element–induced wimpy testis (PIWI) proteins work together with piwi-interacting RNAs (piRNAs) to perform epigenetic regulation and as such hold great potential as biomarkers for pancreatic cancer. PIWIL2 and PIWIL4 are associated with better prognosis, while PIWIL1 and PIWIL3 involvement appears to be associated with carcinogenesis. We aimed to discover PIWIL3- and PIWIL4-modulated piRNAs and determine their potential mechanisms in pancreatic cancer and the clinical implications. PIWIL3 or PIWIL4 was downregulated in pancreatic cancer-derived cell lines or in a non-tumour cell line. Differentially expressed piRNAs were analysed by next generation sequencing of small RNA. Nine fresh-frozen samples from solid human pancreases (three healthy pancreases, three intraductal papillary mucinous neoplasms, and three early-stage pancreatic cancers) were included in the sequencing analysis. Two piRNAs associated with PIWIL3 (*piR-168112* and *piR-162725*) were identified in the neoplastic cells; in untransformed samples, we identified one piRNA associated with PIWIL4 (*pir-366845*). After validation in pancreatic cancer-derived cell lines and one untransformed pancreatic cell line, these piRNAs were evaluated in plasma samples from healthy donors (n = 27) or patients with pancreatic cancer (n = 45). Interestingly, *piR-162725* expression identified pancreatic cancer patients versus healthy donors in liquid biopsies. Moreover, the potential of the serum carbohydrate antigen 19-9 (CA19-9) biomarker to identify pancreatic cancer patients was greatly enhanced when combined with *piR-162725* detection. The enhanced diagnostic potential for the early detection of pancreatic cancer in liquid biopsies of these new small non-coding RNAs will likely improve the prognosis and management of this deadly cancer.

## 1. Introduction

Pancreatic ductal adenocarcinoma (PDAC) is one of the tumours with the highest incidence and aggressiveness in developed countries [1]. PDAC is the third leading cause of death in men and women, and mortality from this disease has increased slowly in the last few years, from 12.1 to 12.7 per 100,000 in men, and from 9.3 to 9.6 per 100,000 in women [1]. By 2030, the incidence of PDAC is expected to surpass that of other malignancies such as breast, prostate, or colorectal cancer [2]. The 5-year survival rate is 50% when tumours are <2 cm in size and close to 100% for tumours <1 cm [3]. However, the probability of detecting tumours <1 cm is low.

There are certain risk factors associated with PDAC occurrence and development. The primary acquired risk factors for PDAC are cigarette smoking (hazard ratio (HR) = 1.74) and high alcohol consumption (HR = 1.1–1.5) [4]. Diabetes has recently been considered a potential early symptom of PDAC, as approximately 30% of PDAC patients were found to have developed the disease [5].

With regard to treatment, findings from the PRODIGE 24 and APACT phase 3 trials support the importance of patient selection for treatment administration and survival [6,7]. As expected, treatment with mFOLFIRINOX (modified fluorouracil, leucovorin, irinotecan, and oxaliplatin) significantly prolonged overall survival (OS) and progression-free survival (PFS) compared to gemcitabine alone (median OS: 54.4 months vs. 35 months, HR = 0.64 (95% confidence interval (CI): 0.48–0.86); median PFS: 21.6 months vs. 12.8 months, HR = 0.58 (95% CI: 0.46–0.73)) [8]. Additionally, a combination of gemcitabine plus nab-paclitaxel significantly prolonged median OS compared to gemcitabine alone, from 36.2 months to 40.5 months (HR = 0.82 (95% CI: 0.68–1.00)), and median PFS increased from 18.8 months to 19.4 months (HR = 0.88 (95% CI: 0.73–1.06)) [7]. Despite these advances, PDAC develops multi-pathway chemoresistance resulting from the interaction between tumour cells, cancer stem cells, and the tumour microenvironment [9]. Indeed, the pancreatic tumour microenvironment, which is composed of pancreatic stellate cells, regulatory T cells (Tregs), tumour-associated macrophages, and myeloid-derived suppressor cells, has immunosuppressive characteristics that regulate proliferation, invasion and metastasis, chemoresistance, and immune evasion [4]. Depending on the location of the primary tumour, the best treatment approach for the management of PDAC is surgical resection performed using the Whipple procedure or its modifications [10].

Unfortunately, the symptoms of PDAC are underestimated and treated on an outpatient basis, which gives tumours time to develop metastases to distant organs. At this point, the tumour becomes unresectable, which drastically reduces the 5-year survival rate, [11,12]. Only 30% of patients present resectable disease at diagnosis [13]. Therefore, novel screening methods based on diagnostic biomarkers are currently needed.

To date, the only biomarker approved by the Food and Drug Administration of USA (FDA) for resectable PDAC is the preoperative levels of carbohydrate antigen 19-9 (CA19-9). CA19-9 is sensitive enough to be considered a good prognostic biomarker for PDAC, since patients with high levels of the antigen present significantly poorer OS and PFS [14,15]. However, the specificity of this marker is still questioned, since other clinical events such as biliary obstruction can increase CA19-9 serum levels [16], and up to 10% of the population cannot synthesise the antigen [17].

In recent years, non-coding RNA (ncRNA), especially microRNAs (miRNAs) and long non-coding RNAs (lncRNAs), have become tools for the diagnosis, prognosis, and prediction of PDAC. Several miRNAs and lncRNAs have been found to modulate certain pathways associated with cell proliferation, invasion, and metastasis [18]. However, other types of ncRNAs have been less widely studied and could provide new insights into PDAC management. This is the case for piRNAs (PIWI-interacting RNAs). When bound to PIWI (P-element–induced wimpy testis) proteins form the so-called piRNA-induced silencing complex (piRISC), which plays important roles in epigenetic regulation, the silencing of transposable elements, the protection of genome integrity, gametogenesis, and piRNA biogenesis [19]. PiRNAs are generated from specific genomic loci called piRNA clusters [20].

Some piRNAs have been described as acting as tumour suppressor factors and others as oncogenes. It has been reported that *piRNA-36712* acts as a tumour suppressor factor in breast cancer, and the inhibition of this piRNA promotes the invasive phenotype of tumour cells [21]. Another tumour suppressor factor is *piR-823*. When overexpressed, *piR-823* leads to the arrest of proliferation in gastric cancer models [22], and the downregulation of *piR-823*-suppressed proliferation of colorectal cancer cells [23]. The upregulation of *piR-651*, an oncogene, has been described as promoting tumour growth owing to mediation by Cyclin D1 and CDK4 (Cyclin Dependent Kinase 4) in non-small cell lung cancer [24]. Nevertheless, we must not overlook the fact that piRNAs form a complex with PIWI proteins (PIWIL1, PIWIL2, PIWIL3, and PIWIL4) that belong to the Argonaute family (AGO) [25]. PIWI proteins have been associated with several types of cancer due to their ability to mediate apoptosis, cell proliferation, genomic integrity, invasion, and metastasis [26,27]. Our group has recently described the prognostic role of PIWIL1 and PIWIL2 in PDAC. Although the expression of both proteins is scarce in PDAC, PIWIL2 expression exhibited an outstanding prognostic potential for both PFS and OS, and an association was found with the progenitor molecular subtype of PDAC [28]. We have also discovered the link between PIWIL3 and PIWIL4 and epithelial-to-mesenchymal transition (EMT) and tumour cell dedifferentiation status and chemoresistance. Furthermore, high levels of PIWIL4 expression in PDAC samples were associated with more favourable outcomes [29]. In the present research, we aim to elucidate which piRNAs are regulated by those PIWI proteins with high expression in PDAC such as PIWIL3 or PIWIL4 and determine the potential clinical usefulness of these proteins for the management of PDAC patients.

## 2. Experimental Section

### 2.1. Cell Lines and Cell Culture

The following human PDAC-derived cell lines were purchased and cultured according to the American Type Culture Collection (ATCC, Manassas, Virginia): PANC 04.03 (ATCC no: CRL-2555), PL45 (ATCC no: CRL-2558), BxPC-3 (ATCC no: CRL-1687), and one untransformed human pancreatic ductal epithelial cell line, hTERT-HPNE (ATCC no: CRL-4023). RWP1 (Cellosaurus no: CVCL_4373) and PANC-1 (ATCC no: CRL-1469) were kindly provided by Dr. Fatima Gebauer (CRG, Barcelona, Spain). The RWP-1 and PANC-1 cells were cultured in an RPMI medium supplemented with 10% foetal bovine serum and 1% penicillin-streptomycin (P/S) according to routine practice. All cell lines were maintained at 37 °C in a humidified atmosphere with 5% CO_2_.

### 2.2. Patient Samples and Public Databases

From 2017 to 2019, a total of 20 blood samples were collected from PDAC patients before surgery at the Hepato-Pancreato-Biliary surgical unit of Fundacion Jimenez Diaz University Hospital (Madrid, Spain) with the approval of the research ethics and institutional review boards (Act Number 19/16 on 15 November 2016). All samples were obtained before any neoadjuvant treatments were administered, and none of the patients had concomitant tumours. In addition, 15 healthy volunteers from the same hospital were enrolled as controls; these were screened to ensure that none had a history of cancer and that they had a similar sex distribution and age range as the PDAC samples. Additionally, 25 PDAC plasma samples and 12 plasma samples from healthy donors were provided by the Aragon Health Sciences Institute (Zaragoza, Spain) as part of the Biobank of Aragon and were processed following standard operating procedures with the approval of the research ethics and institutional review boards. In addition, nine different fresh-frozen human samples were provided by the BioBank of University Hospital Clinico San Carlos (Madrid, Spain) with the approval of the research ethics and institutional review boards (no. 17/091-E on 10 March 2017): pancreatic ductal adenocarcinomas (n = 3), intraductal papillary mucinous neoplasms (n = 3), and healthy untransformed pancreatic tissues (n = 3). In this study, neither *pir-366845*, *piR-162725*, nor *piR-168112* expression affected the decision-making in clinical treatment strategies. The TCGA-PanCancer Atlas database, composed of 184 tumour samples, and the Genotype-Tissue Expression (GTEx) database (https://gtexportal.org (accessed on 20 June 2022)), comprising 171 normal samples, were used to determine which of the transcripts associated with *piR-162725* exhibited a positive or negative correlation with PIWIL3 expression. The TCGA dataset was analysed using the cBioPortal to address gene expression and to calculate the Spearman correlation coefficients with PIWIL3 expression [30,31].

### 2.3. RNA Interference, Western Blotting, and Immunocytochemistry

**Figure 1:**
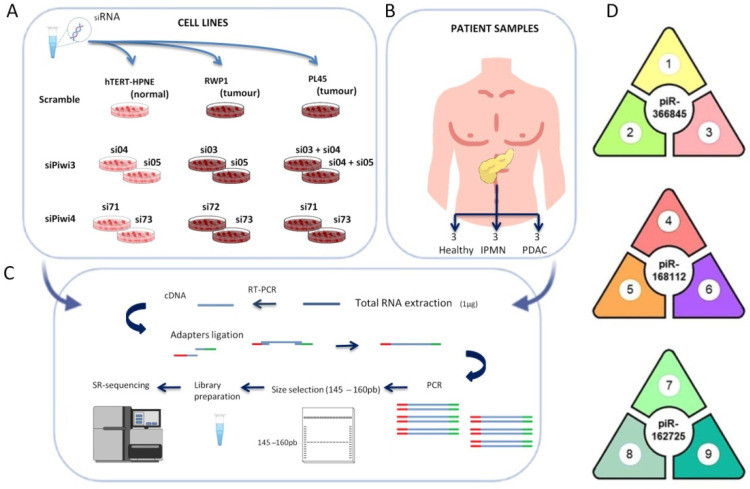
Small RNA sequencing of modified human pancreatic cancer and normal cell lines and human pancreatic tissues revealed three differentially expressed piRNAs. (**A**) Schematic representation of the procedure for PIWIL3 or PIWIL4 downregulation. Here, two pancreatic tumour cell lines and one non-tumour cell line were transfected with two independent siRNA sequences to downregulate PIWIL3 or PIWIL4. As a control, all cell lines were transfected with a scramble siRNA. (**B**) RNA from fresh-frozen human tissues obtained from pancreatic ductal adenocarcinomas (n = 3), intraductal papillary mucinous neoplasms (n = 3), and healthy pancreatic tissues (n = 3) were included in the NGS of small RNA. (**C**) cDNA was obtained from the total RNA isolated from all samples. After adapter ligation by PCR, the fragments were loaded onto a polyacrylamide gel, and bands from 145–160 bp were excised and purified to continue with small RNA library preparation. Sequencing was performed on a MiniSeq Platform (Illumina). (**D**) Three different piRNAs were commonly found in three different data integrations: 1: healthy pancreas tissues vs. PL45 and RWP1 scramble; 2: hTERT-HPNE scramble vs. PL45 and RWP1 scramble; 3: hTERT-HPNE downregulated for PIWIL4 vs. hTERT-HPNE scramble; 4: RWP1 and PL45 downregulated for PIWIL3 vs. hTERT-HPNE downregulated for PIWIL3; 5: RWP1 and PL45 downregulated for PIWIL3 vs. PDAC and intraductal papillary mucinous neoplasm (IPMN) pancreatic samples; 6: RWP1 and PL45 downregulated for PIWIL3 vs. RWP1 and PL45 scramble; 7: RWP1 and PL45 scramble vs. IPMN pancreatic samples; 8: RWP1 and PL45 scramble vs. hTERT-HPNE scramble; 9: RWP1 downregulated for PIWIL3 vs. RWP1 downregulated for PIWIL4; NGS: Next Generation Sequencing.

To isolate the RNA and perform Next Generation Sequencing (NGS) of the small RNA, previously downregulated PIWIL3 or PIWIL4 samples studied by Li et al. were used [29]. Here, the levels of PIWIL3 or PIWIL4 were downregulated in the PDAC-derived cell lines, PL45 and RWP1, and the normal cell line, hTERT-HPNE, using the methods and siRNAs described in Supplementary Table S1 [29]. Subsequently, PIWIL3 or PIWIL4 downregulation was confirmed by Western blot for PL45 and RWP1, and by immunocytochemistry in the case of the untransformed cell line hTERT-HPNE (Figure 1) [29].

### 2.4. Small RNA Isolation, Library Preparation, Small RNA Sequencing, and Bioinformatic Analysis

The total RNA was extracted using the TRIzol reagent (Invitrogen; ThermoFisher Scientific, Inc., Waltham, MA, USA) according to the manufacturer’s protocol. The RNA was quantified via absorbance spectrophotometry on a Nanodrop 2000 instrument (ThermoFisher Scientific, Inc., Waltham, MA, USA) and via RiboGreen dye fluorescence on a Qubit fluorometer (Life Technologies, Carlsbad, CA, USA). The RNA integrity was assessed by electrophoresis using a Total RNA Pico chip on Bioanalyzer 2100 (Agilent, Santa Clara, CA, USA). The RNA aliquots were stored at −80 °C. Thus, 1 μg of RNA was used to generate small RNA libraries using reagents and methods contained in the TruSeq Small RNA Sample Prep Kit version 2 (Illumina, San Diego, CA, USA) for the NGS. Briefly, T4 RNA ligase was used to ligate RA5 and RA3 RNA oligonucleotides to the 5′ and 3′ ends of the RNA, respectively. PCR amplification was performed based on the upper and lower limits recommended by the kits. The adapter-ligated RNA was reverse-transcribed using an RTP primer, and the resulting cDNA was amplified in an 11-cycle PCR that used RP1 and indexed RP1 primers. Then, the libraries were run on a 6% TBE Gel for 55 min at 140 V, and bands between 140 and 160 bp were excised. These gel pieces were fragmented into smaller pieces and incubated overnight in ultrapure water. Subsequently, the DNA fragments were precipitated in ethanol and solubilised in 11 µL of ultrapure water. The quality of the DNA fragments generated was confirmed using a high-sensitivity DNA analysis kit (Agilent, Santa Clara, CA, USA) on a Bioanalyzer 2100 instrument. Quadruplexed sequencing of the libraries to generate single-end reads of 50 bp was performed on a MiniSeq 2000 Platform (Illumina, San Diego, CA, USA) using MiniSeq clustering and sequencing reagents (version 2.0).

The reads from the FASTQ files were aligned, annotated, and counted using the sRNAnalyzer pipeline [32]. We used Bowtie (1.1.2) for alignment to the hg19 human genome. Annotation of the small RNA was performed using updated versions of the main databases, i.e., miRBase (14 January 2019), piRBase (14 January 2019), snoRNABase (20 December 2018), and lncipedia (20 January 2018), as well as the databases provided by sRNAnalyzer within the MainDBs subset. The maximum mismatch allowance was one for all databases. The sRNAnalyzer pipeline was run using the following parameters: stop-oligo = true, min-length = 15, and the kit: Illumina MiniSeq. The differential expression analysis of piRNAs was performed using DESeq2 from the bioconductor repository [33]. Significant piRNAs (adjusted *p*-value < 0.05) in each pairwise comparison were extracted and annotated as up- and downregulated with regard to the corresponding classes.

### 2.5. Quantification of piRNAs in Blood Samples by Real-Time PCR

The blood samples were collected in EDTAK2 tubes (BD Vacutainer^®^, Franklin Lakes, NJ, USA) and centrifuged at 1600× *g* for 10 min at room temperature. The supernatant was carefully transferred into 2-mL tubes and centrifuged at 16,000× *g* for 10 min at 4 °C. The plasma was aliquoted and stored at −80 °C for small RNA isolation. The small RNA was extracted from 800 mL of plasma with the miRNeasy Serum/Plasma kit (Qiagen, Hilden, Germany) following the manufacturer’s protocol. To allow for the normalisation of sample-to-sample variation in the RNA isolation step, 25 fmol of a synthetic RNA oligonucleotide, *cel-miR-39* (UCACCGGGUGUAAAUCAGCUUG) (Assay ID: MC10956; Life Technologies, Carlsbad, CA, USA) was introduced in each denatured sample as previously reported [29]. The reverse transcription reaction was carried out with a TaqMan MicroRNA Reverse Transcription Kit (Life Technologies, Carlsbad, CA, USA) for each of the following customised primers: *hsa-piR-168112* (AGCAGAGTGGCGCAGCGGAAGCGTGCTGGGCCCT), *hsa-piR-162725* (GCCCGGCTAGCTCAGTCGGTAGAGCATGCGACTC), *cel-miR-39* (Assay ID: 000200; Life Technologies, Carlsbad, CA, USA), and *RNU6B* (Assay ID: 001093; Life Technologies, Carlsbad, CA, USA). A real-time PCR was carried out with the TaqMan Universal PCR Master Mix, no AmpErase UNG (Life Technologies, Carlsbad, CA, USA) following the manufacturer’s instructions in the Applied Biosystems 7500 Sequence Detection System. The expression of piRNAs in the PDAC cell lines was normalised to *RNU6B* expression (Assay ID: 001093; Life Technologies, Carlsbad, CA, USA). The expression of piRNAs in the human plasma samples was analysed with the 2−ΔΔCT method. For this, ΔCT of each piRNA was referred to as *cel-miR-39* expression for each sample. Then, ΔΔCT of each piRNA was referred to as the expression of the same piRNA in the pancreatic cell line with the highest piRNA expression as a normaliser. Therefore, *piR-168112* and *piR-162725* were normalised according to the expression in the PANC 04.03 cell line.

### 2.6. Statistical Analysis

To analyse each piRNA from the human plasma samples statistically, the normal distribution of expression was determined by the Kolmogorov–Smirnov test. The statistical differences between the groups of plasma samples (tumour vs. healthy) were assessed using the Student’s T-test for parametric samples. Statistical analyses between PDAC patients identified by each of the biomarkers were performed as related samples analysis with the Wilcoxon rank-sum test. The PFS and OS curves according to *piR-168112* or *piR-162725* were analysed with Kaplan–Meier curves, and survival was analysed by the log-rank test. The Cox proportional hazards model was used to assess the hazard ratios and confidence intervals of the clinico-pathological variables of patients included in the study. Only statistically significant variables found in the univariate analysis were included in the multivariate analysis. All statistical analyses were performed with IBM SPSS statistics 20.0. We further dissected potential target transcripts associated with *piR-162725* as a function of their base complementarity. Kyoto Encyclopedia of Genes and Genomes (KEGG) pathway analysis together with Gene Ontology analysis was used to predict the potential molecular pathways [34]. KEGG analysis showed that a *p* < 0.05 denoted statistical significance. Furthermore, the KEGG pathway analysis was conducted on the “cluster profiler R” package [35]. Adjusted *p*-values < 0.05 were considered statistically significant.

## 3. Results

### 3.1. Differentially Expressed piRNAs Associated with PIWIL3 Exhibit a Transforming Effect, whereas a piRNA Associated with PIWIL4 Had Protective Effects

PIWIL3 or PIWIL4 were individually downregulated with two different validated siRNA sequences in two PDAC derived cell lines (PL45 and RWP1) that presented the highest expression levels of both proteins according to previous reports [29]. Two independent combinations with two different siRNAs were necessary to downregulate PIWIL3 in Pl45 cells due to its overexpression (Figure 1A) [29]. In addition, PIWIL3 or PIWIL4 was downregulated as previously described [29] in one non-tumour pancreatic cell line (hTERT-HPNE) (Figure 1A). As a control, all three cell lines were transfected with a negative scramble control that did not interfere with either PIWIL3 or PIWIL4 expression [29]. As a validation cohort of human pancreatic cells and to supplement the biostatistical analyses, we also included nine different fresh-frozen human samples in this high-throughput study: pancreatic ductal adenocarcinomas (n = 3), intraductal papillary mucinous neoplasms (n = 3), and healthy untransformed pancreatic tissues (n = 3) (Figure 1B). These 24 samples were processed to isolate their total RNA and to prepare all libraries for the NGS of small RNA (Figure 1C).

We characterised the small non-coding transcriptome by NGS and then conducted an integration analysis to discover the differentially expressed piRNAs between tumour-derived and non-tumour-derived pancreatic cell lines according to PIWIL3 or PIWIL4 downregulation, following a validation with piRNAs obtained in tumour and non-tumour tissue samples. The analyses rendered between 3995 and 13,956 total piRNAs; of them, 94 significant differentially expressed piRNAs were found in several integration analyses ( Supplementary Table S1). Interestingly, 89 piRNAs were significantly differentially expressed between the non-tumour cell line (hTERT-HPNE) transfected with scramble and human samples from pancreatic ductal adenocarcinoma (PDAC). In addition, 52 significant differentially expressed piRNAs were identified after the integration analysis of hTERT-HPNE transfected with scramble and IPMN samples. Furthermore, we found a large number of differentially expressed piRNAs when comparing tumour cell lines (RWP1 and PL45) downregulated for PIWIL3 or PIWIL4 against IPMN samples (n = 94) or against human PDAC samples (n = 22) ( Supplementary Table S1).

The data integration and analysis after validation with human tissues revealed a highly significant association between one common piRNA and untransformed cells or tissues (*pir-366845*) and a high statistical significance for two common piRNAs with tumour samples (*piR-168112* and *piR-162725*) (Figure 1D). *Pir-366845* was significantly upregulated, not only in healthy pancreatic tissues compared to both tumour cell lines (Log2 fold change = −23.62; adjusted *p*-value = 5.94 × 10^−7^), but also in the normal cell line (hTERT-HPNE) compared to both tumour cell lines (Log2 fold change = −24.14; adjusted *p*-value = 1.74 × 10^−5^). These results suggest a role for *pir-366845* as a protective factor associated with normal untransformed tissues. In addition, *pir-366845* was downregulated in the healthy cell line upon PIWIL4 silencing compared to the control cell line transfected with scramble (Log2 fold change = −24.14; adjusted *p*-value = 1.74 × 10^−5^). This fact suggested that *pir-366845* is under PIWIL4 modulation.

In contrast, *piR-168112* expression was lower in tumour cell lines after PIWIL3 silencing than in the same cell lines transfected with scramble (Log2 fold change = −25.58; adjusted *p*-value = 6.32 × 10^−10^). This result suggested that *piR-168112* could be regulated by PIWIL3. *PiR-168112* was also downregulated in the PDAC and IPMN samples compared to tumour cell lines (Log2 fold change = –26.81; adjusted *p*-value = 1.31 × 10^−15^). Furthermore, the expression of *piR-168112* was even lower in the normal, untransformed cell line after PIWIL3 silencing compared to tumour cell lines after PIWIL3 silencing, the latter showing higher expression (Log2 fold change = −27.10; adjusted *p*-value = 7.52 × 10^−11^). This result suggested that *piR-168112* has tumorigenic potential.

Finally, *piR-162725* was significantly downregulated in the RWP1 tumour cell line after PIWIL3 silencing compared to the same tumour cell line after PIWIL4 silencing (Log2 fold change = −24.44; adjusted *p*-value = 1.63 × 10^−5^). This fact supports the notion that *piR-162725* could be driven by PIWIL3. In addition, *piR-162725* was significantly downregulated in the normal, untransformed cell line compared to both tumour cell lines (Log2 fold change = −15.04; adjusted *p*-value = 0.0091). Curiously, *piR-162725* was also upregulated in both tumour cell lines compared to pre-malignant IPMN (Log2 fold change = 12.13; adjusted *p*-value = 0.0014). These two findings indicate that *piR-162725* could play an oncogenic role in pancreatic cancer.

**Figure 2:**
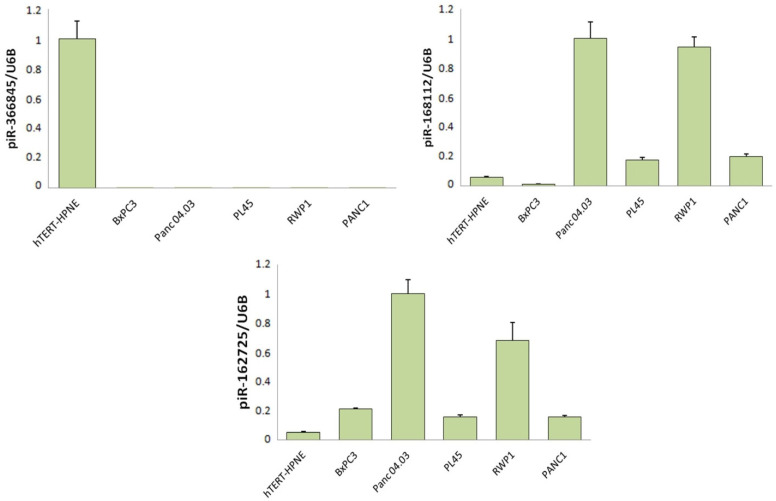
Validation of the three piRNAs obtained from the NGS of small RNA in untransformed and pancreatic-cancer-derived cell lines. Statistically significant piRNAs obtained from data integration and analyses from the NGS of small RNA (*pir-366845*, *piR-168112*, and *piR-162725*) were evaluated in one untransformed human pancreatic cell line (hTERT-HPNE) and in five human pancreatic-cancer-derived cell lines. Graphs show piRNA fold-change expression normalised to U6B small nuclear RNA gene (RNU6B) expression. NGS: Next Generation Sequencing.

Since these three piRNAs were obtained from high-throughput techniques, we validated them in a panel of five PDAC-derived cell lines and one untransformed human pancreatic-duct-derived cell line immortalised with human telomerase (hTERT-HPNE) accordingly. For this aim, we designed assays for mRNA determination by real-time PCR according to the piRNA sequences (see Experimental Section). All PDAC-derived cell lines showed a lack of expression of *pir-366845*, while the untransformed cell line presented the highest *pir-366845* expression (Figure 2, top-left). We expected these results, since data integration and analysis of the sequencing experiments revealed the upregulation of *pir-366845* in the healthy untransformed samples compared to tumour cell lines. In contrast, the expression of *piR-168112* and *piR-162725* was higher in tumour cell lines compared to the untransformed cell line, which was in accordance with the NGS analyses. Interestingly, the pattern of expression of both piRNAs in the tumour cell lines was similar except for the BxPC-3 cell line (Figure 2, top-right). The expression of *piR-168112* and *piR-162725* was highest in the Panc 04.03 and in the RWP1 tumour cell lines (Figure 2, top-right and bottom).

Thus, *piR-162725* seems an optimal candidate biomarker to differentiate between tumour and non-tumour samples.

### 3.2. High Expression of piR-162725 in Human Plasma Samples Differentiates Tumour Samples from Healthy Samples

Since piRNAs are involved in several tumour types, we aimed to evaluate these piRNAs in PDAC patients. Furthermore, as piRNAs are small in size and highly stable in biological fluids, this raised the question as to whether these piRNAs could be used to distinguish between tumour and healthy samples based on their expression profile in plasma samples from PDAC. To this end, we obtained 15 plasma samples from healthy donors and 20 plasma samples from PDAC patients of the hepato-pancreato-biliary surgical unit at our institution (referred to as the institutional cohort (IC)).

**Scheme 1:**
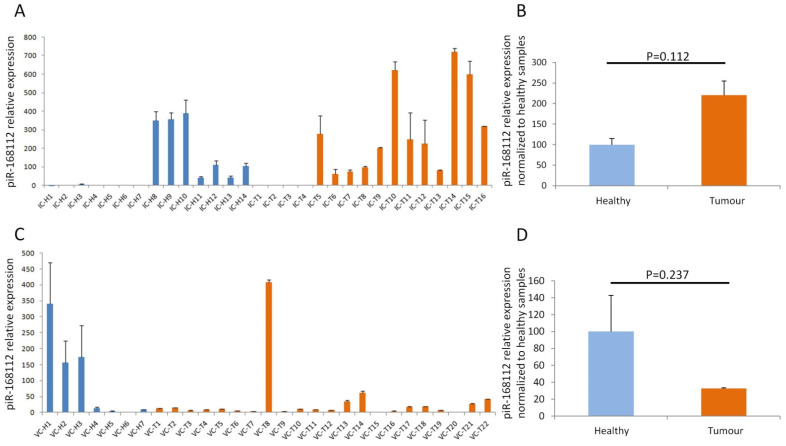
Expression of *piR-168112* in human plasma samples. (**A**) Relative *piR-168112* expression in plasma samples from 14 healthy donors and 16 PDAC patients recruited institutionally. (**B**) Statistical analysis of *piR-168112* expression in plasma samples from our institutional cohort grouped by their status, i.e., healthy or tumour. (**C**) Relative *piR-168112* expression in plasma samples from seven healthy donors and 22 PDAC patients from a validation cohort. (**D**) Statistical analysis of *piR-168112* expression in plasma samples from the validation cohort grouped by healthy or tumour status. IC-H: Institutional cohort healthy samples. IC-T: Institutional cohort tumour samples. VC-H: Validation cohort healthy samples. VC-T: Validation cohort tumour samples. *p*-values < 0.05 were considered statistically significant.

As *pir-366845* was associated with the healthy, untransformed phenotype, as revealed from the NGS of small RNA approach, and was downregulated in all PDAC-derived cell lines, we decided not to evaluate *pir-366845* in the PDAC plasma samples since tumour phenotyping in clinical practice is easier when it is based on overexpression than on downregulation. Therefore, we analysed the other piRNAs, *piR-168112* and *piR-162725*, in the plasma samples since these were overexpressed in tumour samples and PDAC-derived cell lines. *PiR-168112* expression was evaluated in all samples of the IC; however, its expression was undetermined in some samples, and these cases were not included in the analysis. The expression profile of *piR-168112* in healthy and tumour samples showed a differential expression pattern, whereas more healthy samples expressed minimal *piR-168112* levels (Scheme 1A). We then grouped the plasma samples according to their origin, and differences in expression were observed between the healthy samples (relative expression = 100 ± 15) and the tumour samples (relative expression = 220 ± 34). However, statistical analysis revealed no significant differences between the samples (*p* = 0.112) (Scheme 1B).

To verify this result, we evaluated *piR-168112* expression in a validation cohort composed of 12 plasma samples from healthy donors and 25 PDAC plasma samples from the Spanish Biobank Network (referred to as the validation cohort (VC)). The expression of this piRNA in some samples was undetermined; as in previous analyses, these samples were not included, and most samples expressed very low levels of *piR-168112* (Scheme 1C). Median *piR-168112* expression in the tumour samples was even lower (relative expression = 32 ± 1) compared to the healthy plasma samples (relative expression = 100 ± 43) (*p* = 0.237; Scheme 1D). Unfortunately, the expression profile of *piR-168112* between the IC and VC differed (Scheme 1B,D). The tumour samples from our institution expressed a two-fold higher change than the healthy samples, while the tumour samples from the validation set showed three times lower expression than the healthy samples (Scheme 1B,D). These results reflected that *piR-168112* may not be a good candidate for use as a diagnostic biomarker for PDAC.

**Figure 3:**
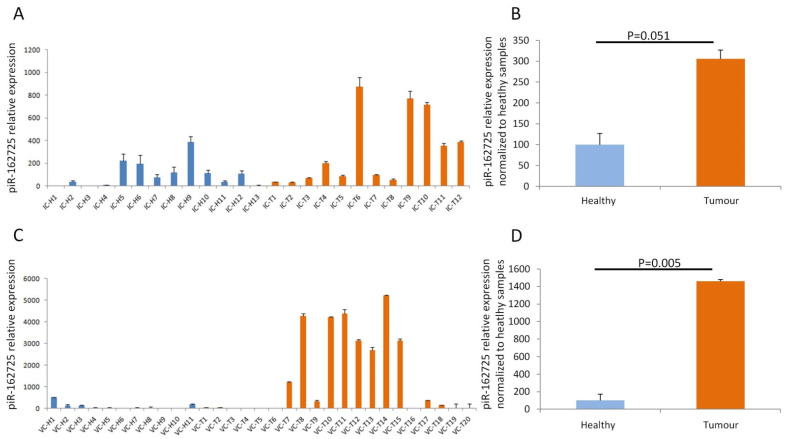
Expression of *piR-162725* in human plasma samples. (**A**) Relative *piR-162725* expression in plasma samples from healthy donors and PDAC patients recruited institutionally. (**B**) Statistical analysis of *piR-162725* expression in plasma samples from our institutional cohort grouped by their status, i.e., healthy or tumour. (**C**) Relative *piR-162725* expression in plasma samples from healthy donors and PDAC patients from a validation cohort. (**D**) Statistical analysis of *piR-162725* expression in plasma samples from the validation cohort grouped by healthy or tumour status. IC-H: Institutional cohort healthy samples. IC-T: Institutional cohort tumour samples. VC-H: Validation cohort healthy samples. VC-T: Validation cohort tumour samples. *p*-values < 0.05 were considered statistically significant.

Subsequently, we evaluated the expression of *piR-162725* in both the IC and VC of PDAC and healthy samples. The evaluation of *piR-162725* in our IC revealed differential expression among tumour samples compared to healthy samples (Figure 3A). When we grouped the samples by their origin, the tumour samples expressed a three-fold higher change (relative expression = 305 ± 21) than the healthy samples (relative expression = 100 ± 26). The statistical analysis of the IC revealed a strong trend toward significance between the expression of tumour and healthy samples (*p* = 0.051) (Figure 3B). The evaluation of *piR-162725* in the healthy samples from the VC showed very low levels compared to the tumour samples (Figure 3C). Interestingly, the median expression of *piR-162725* in the tumour samples was 14-fold higher (relative expression = 1460 ± 22) than the median expression of the healthy samples (relative expression = 100 ± 72). The statistical analysis between the tumour and healthy samples of the VC revealed highly significant differences between both groups (*p* = 0.005) (Figure 3D). The results obtained here suggest that *piR-162725* has high potential as a biomarker for the identification of PDAC patients by simple blood testing.

### 3.3. High Expression of piR-162725 in Human Plasma Samples Increases the Sensitivity of CA19-9 as a Liquid Biopsy Biomarker to Identify PDAC Patients

**Figure 4:**
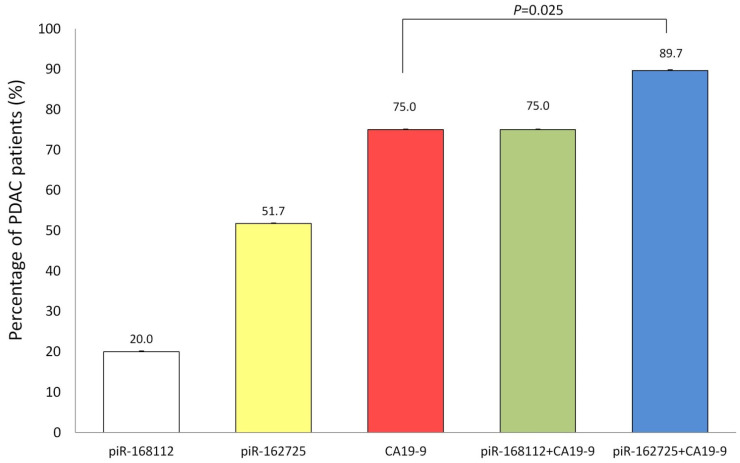
Expression of *piR-162725* in human plasma samples enhances the diagnostic potential of CA19-9. The bar graph represents the percentage of PDAC patients identified by any factor individually (*piR-168112* (white), *piR-162725* (yellow)*,* or CA19-9 (red)) or in combination with CA19-9 (*piR168112*+CA19-9 (green) or *piR162725*+CA19-9 (blue)). *p*-value < 0.05 was considered statistically significant.

Since CA19-9 is the most important and widely used clinical tumour marker worldwide for patients with pancreatic cancer, we aimed to compare the identification potential of *piR-162725* with CA19-9 levels in PDAC patients. We therefore categorised levels of CA19-9 of our PDAC plasma samples using 37 U/mL as a cut-off point according to Kim et al. [16]. Concerning our piRNAs, the median expression of healthy samples was considered the best cut-off point to differentiate between positive and negative *piR-168112* or *piR-162725* expression. According to our PDAC plasma samples, CA19-9 enables the identification of 75% of PDAC patients (Figure 4). In both piRNAs, PDAC identification potential was always below that of CA19-9 expression. As expected, *piR-168112* only identified 20% of PDAC cases, and combining it with CA19-9 did not increase the accuracy of CA19-9 (Figure 4). However, not only could *piR-162725* positiveness identify half of the PDAC cases per se, as well as 14% of CA19-9 negative cases, but also the combination of *piR-162725* and CA19-9 increased the sensitivity to 89.7%, which increases CA19-9 sensitivity by 15%. The statistical analysis of the comparison between the percentage of PDAC cases identified by CA19-9 alone and when combined with *piR-162725* revealed statistically significant differences (*p* = 0.025) (Figure 4). Therefore, this result supports the role of *piR-162725* as a potential clinical biomarker for the identification of PDAC patients when used in combination with CA19-9 to increase its performance potential.

### 3.4. High Expression of piR-162725 or piR-168112 Is Not Associated with Patient Outcome

**Figure 5:**
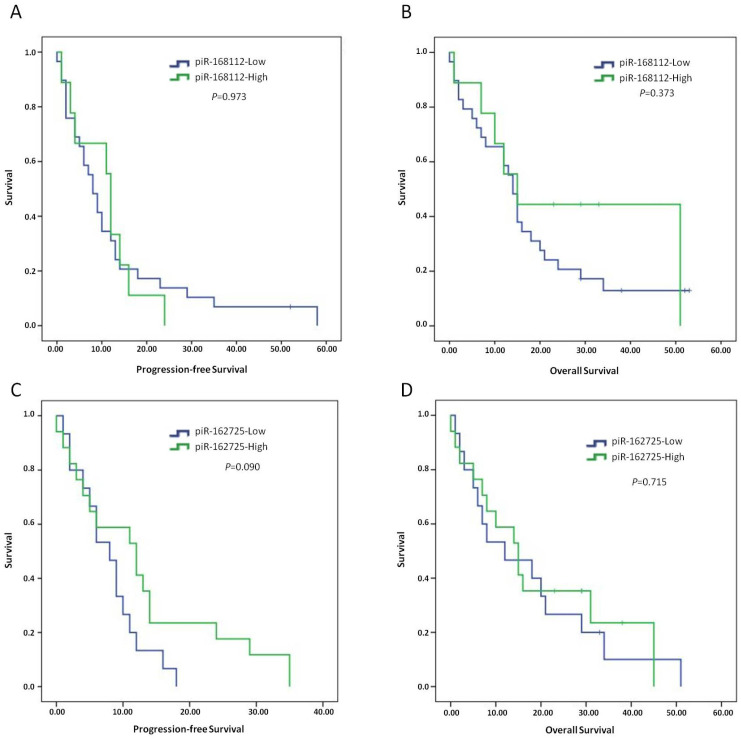
Survival curves according to the expression levels of both tumourigenic piRNAs. (**A**) Kaplan–Meier curve for progression-free survival according to *piR-168112* expression. (**B**) Kaplan–Meier curve for overall survival according to *piR-168112* expression. (**C**) Kaplan–Meier curve for progression-free survival according to *piR-162725* expression. (**D**) Kaplan–Meier curve for overall survival according to *piR-162725* expression. Statistical analyses were performed using the log-rank test. *p*-values < 0.05 were considered statistically significant.

To further characterise both piRNAs associated with tumorigenesis, we obtained clinical information on all the PDAC patients recruited for the present study as well as histopathologic information on their tumours. Then, by using the same cut-off points of the piRNAs from the previous section to discriminate between the high and low expression levels of both piRNAs, we were able to plot survival curves and conduct statistical analyses. None of the piRNAs was significantly associated with either disease progression or OS (Figure 5). *PiR-168112* was not associated with PFS (median high expression = 12 months; 95% CI = 10-6-13-3 vs. median low expression = 8 months; 95% CI = 5.3–10.6; *p* = 0.937) (Figure 5A). The median OS according to *piR-168112* also did not differ significantly in terms of its expression (median high expression = 15 months; 95% CI = 6.2–23.7 vs. median low expression = 14 months; 95% CI = 11.8–16.1; *p* = 0.373) (Figure 5B). Concerning *piR-162725*, a strong trend toward significance was found for PFS (median high expression = 12 months; 95% CI = 4.0–19.9 vs. median low expression = 8 months; 95% CI = 4.9–11.0; *p* = 0.090) (Figure 5C); and OS according to *piR-162725* expression did not reach significance (median high expression = 15 months; 95% CI = 8.3–21.6 vs. median low expression = 12 months; 95% CI = 0–25.8; *p* = 0.715) (Figure 5D). Therefore, neither *piR-168112* nor *piR-162725* could be considered as prognostic biomarkers for PDAC patients.

Subsequently, we applied a proportional hazard model for all the available clinico-pathological information concerning the patients and their tumours (Table 1). Here, we observed that tumour stage (HR = 2.467; 95% CI = 1.137–5.353; *p* = 0.022) and metastatic disease at diagnosis (HR = 2.062; 95% CI = 1.047–4.061; *p* = 0.036) were associated with shorter PFS. However, neither of these variables remained significant in the multivariate analysis. Nonetheless, in the univariate analysis for OS, tumour stage (HR = 5.545; 95% CI = 1.909–16.109; *p* = 0.002) and metastatic disease at diagnosis (HR = 5.840; 95% CI = 2.386–14.298; *p* = 0.001) presented higher levels of significance compared to a univariate analysis for PFS. Furthermore, tumour size (HR = 3.302; 95% CI = 1.134–9.616; *p* = 0.029) also appeared to be statistically significant. In the multivariate analysis for OS, the only variable that remained significant was metastatic disease at diagnosis, which we had envisaged since these cases do not benefit from surgical resection, thereby drastically reducing patient survival.

### 3.5. The Potential piR-162725/PIWIL3 Complex Is an Undercover Modulator of Tumourigenic Signalling Pathways

**Figure 6:**
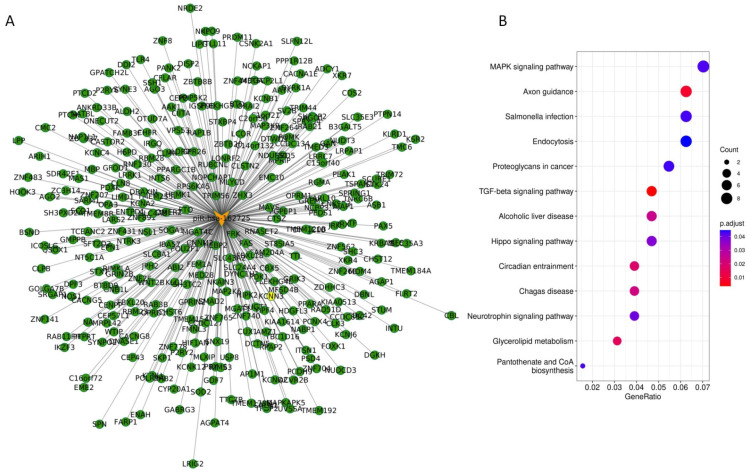
KEGG pathway analysis of *piR-162725* highlights its potential as a modulator of the MAPK signalling pathway. (**A**) *PiR-162725*-mediated KEGG pathway network showing putative mRNA interaction with *piR-162725.* (**B**) Dot plot of the top 13 KEGG pathways which were enriched in *piR-162725* potentially modulated transcripts.

Since piRNAs are involved in the epigenetic regulation of several transcripts, and given that *piR-162725* seems to be the only factor with a clear tumourigenic and diagnostic potential relative to the other two piRNAs of our study and as a complement to CA19.9, we set out to determine which targets could be modulated by *piR-162725* in tumour cells. A prediction model based on sequence complementarity rendered a total of 317 transcripts potentially modulated by *piR-162725* (Figure 6A). Interestingly, KEGG enrichment analysis revealed that several transcripts statistically associated with three signalling pathways related to cell proliferation might be regulated by *piR-162725*: Mitogen-Activated Protein Kinase (MAPK) (eight transcripts; GeneRatio:0.07; adjusted *p*-value = 0.05); Transforming Growth Factor Beta (TGF-beta) (six transcripts; GeneRatio:0.047; adjusted *p*-value < 0.01), and Hippo Pathway (four transcripts; GeneRatio:0.047; adjusted *p*-value = 0.03) (Figure 6B).

In an attempt to narrow the number of transcripts modulated by *piR-162725*, we took into consideration our bioinformatic-based results that suggest that *piR-162725* may work together with PIWIL3, forming a potential *piR-162725*/PIWIL3 complex to modulate several transcripts positively or negatively [36]. To do this, we used the TCGA-PanCancer Atlas database and the Genotype-Tissue Expression (GTEx) database composed of 184 tumour samples and 171 normal samples to confirm that both *piR-162725* and PIWIL3 were highly expressed in tumour tissues, and to discover which of the transcripts associated with *piR-162725* exhibited a positive or negative correlation with PIWIL3 expression. A negative correlation with PIWIL3 could indicate transcript repression by the potential *piR-162725*/PIWIL3 complex. In contrast, a positive correlation with PIWIL3 may suggest the stabilisation of transcripts by the potential *piR-162725*/PIWIL3 complex that enables translation. Here, we observed 170 transcripts that were downregulated, 146 upregulated, and only one transcript with no change ( Supplementary Table S2). Interestingly, only four transcripts were downregulated in PDAC samples while showing a significant negative correlation with PIWIL3: RIMS3 (ρ = −0.148; *p* = 0.047), P2RY6 (ρ = −0.165; *p* = 0.027), SHC3 (ρ = −0.168; *p* = 0.025), and SH3BP2 (ρ = −0.187; *p* = 0.012) ( Supplementary Table S2). These factors may not be expressed in PDAC samples because the potential *piR-162725*/PIWIL3 complex must be present, and it is involved in their downregulation. Another factor that exhibited a negative correlation with PIWIL3 and was overexpressed in the PDAC samples was P2RY2 (ρ = −0.196; *p* = 0.009) ( Supplementary Table S2). This suggests that the lack of the potential *piR-162725*/PIWIL3 complex may allow the upregulation of P2RY2. Concerning the transcripts that correlated positively with PIWIL3, RAB3B (ρ = 0.152; *p* = 0.043) and SOGA1 (ρ = 0.213; *p* = 0.004) were upregulated, and those transcripts that were downregulated together with PIWIL3 downregulation were TBC1D16 (ρ = 0.149; *p* = 0.046) and CACNA1E (ρ = 0.160; *p* = 0.032) ( Supplementary Table S2). We hypothesise that upregulated transcripts may be stabilised by the potential *piR-162725*/PIWIL3 complex and may act as tumour susceptibility factors, while downregulated transcripts in PDAC samples together with a lack of *piR-162725* andPIWIL3 expression may indicate a potential protective function of these transcripts.

## 4. Discussion

PDAC is an extremely lethal malignancy for which early diagnosis is crucial to increase patient survival. New molecular biomarkers will therefore play an important role in the future management of this neoplasm. Next-generation sequencing brings high-throughput results and creates an opportunity to discover several mutations by whole genome analysis, to reveal differentially expressed genes by transcriptome analysis, and to uncover other novel factors such as non-coding RNAs by NGS. The evaluation and clinical characterisation of non-coding RNAs has made it possible to research lncRNAs, miRNAs, piRNAs, and other factors such as snoRNA in pancreatic cancer [37,38]. PiRNAs have several similarities to miRNAs, which have emerged as high-potential biomarkers for cancer diagnosis based on liquid biopsy with the increasing power and availability of RNAseq techniques [39]. In fact, piRNAs represent 1.31% of all mappable small RNA counts in human plasma [40]. Our strategy began with the evaluation of PIWI proteins, which resulted in PIWIL3 and PIWIL4 being the most highly expressed in PDAC [29]. In the present article, we used NGS of small RNA to analyse the piRNA profile of PDAC-derived cell lines and human samples and other untransformed cell lines and healthy human samples to discover differentially expressed piRNAs after PIWIL3 or PIWIL4 downregulation. Data integration and analysis revealed three common and significant differentially expressed factors: *pir-366845*, *piR-168112*, and *piR-162725*. Of these, *piR-162725* showed the potential to identify plasma samples from PDAC patients. The findings from studies that report piRNAs in blood samples from PDAC have been rather limited. As far as we are aware, only one study performed with six plasma samples from borderline resectable and metastatic stages discovered two piRNAs: *piR-016658* and *piR-001311* [41]. Other authors analysed piRNAs from serum samples from healthy donors, IPMN, and PDAC by NGS and obtained an upregulated piRNA profile in tumour samples compared to healthy ones containing *piR-52959*, *piR-53108*, *piR-30690*, *piR-54479*, and *piR-56621*, and a piRNA profile downregulated in tumour samples from patients that contained *piR-54888*, *piR-42185*, *piR-46410*, *piR-58897*, and *piR-43043* [42]. In another NGS study, *piR-017061* appeared downregulated in PDAC samples (n = 6) compared to untransformed pancreatic samples (n = 5), which suggested a protective effect of this piRNA in PDAC [43]. Subsequently, functional experiments revealed that the re-expression of *piR-017061* impaired the proliferation of PDAC tumour cells in vitro and in vivo [44]. A large-scale study with 10,997 tissue samples across 33 cancer types that included pancreatic cancer identified significantly low expression levels of *piR-317* in cancer samples compared to normal tissues, which suggested this piRNA as a potential tumour suppressive factor, and the upregulation of *piR-1945036*, which suggested the oncogenic potential of this piRNA [45]. Indeed, patients with low expression levels of *piR-1945036* presented longer survival rates than those with high expression levels [45]. Unfortunately, in our study the detection of *piR-168112* or *piR-162725* in plasma samples from PDAC samples was not associated with PFS or OS. However, we would have liked to compare our results from the plasma samples with the evaluation of both piRNAs in solid samples from surgical resections at early stages, where we expect higher expression levels and greater sample homogeneity.

To date, the only biomarker approved by the FDA for PDAC are preoperative levels of CA19-9, which have a sensitivity of 79–81% for diagnosing pancreatic cancer in symptomatic patients [46]. This sensitivity surpasses that of other biomarkers such as carcinoembryonic antigen (CEA), carbohydrate antigen 50 (CA-50), and DUPAN-2 [47,48]. However, CA19-9 cannot be used in screening because of its low positive predictive value (0.5–0.9%) and many other limitations such as its poor sensitivity, low levels in the Lewis negative phenotype (5–10% of cases), and high levels associated with obstructive jaundice (10–60% of cases) [49]. Therefore, there is still an unmet clinical need for biomarkers in PDAC management. This need may be met in two ways: by finding new biomarkers with sufficient accuracy, or by upgrading existing biomarkers though combination with other markers. Based on our results, we propose the latter, that is, the addition of *piR-162725* determination in plasma samples to CA19-9, as doing so increases sensitivity significantly, from 75% to 89.7%. Furthermore, we found that *piR-162725* could identify 14% of patients that were negative for CA19-9, which supports its potential role as a diagnostic biomarker However, the association between tumour stages (early and advanced) and the positive expression of *piR-162725* did not reach statistical significance (*p* = 0.444). This low statistical power could be because only 30% of the samples were from early-stage tumours (I/II), while 70% of the samples were from late stages (III/IV). Therefore, further experiments are needed to affirm that *piR-162725* could be an early-stage diagnostic biomarker for PDAC. Moreover, we recognise that specificity has not been determined by the quantification of *piR-162725* in other gastrointestinal tumours or disease conditions such as obstructive jaundice. Nonetheless, our research performed in plasma samples from PDAC has the clinical applicability to improve the diagnosis of this lethal disease. A previous study supported the use of a combination of two markers, IGF-1 and albumin, with CA19-9 to increase the sensitivity to 93.6% in pancreatic cancer [50]. Thus, it could be worthwhile to study an independent cohort and establish whether the differences in sensitivity between the determination of *piR-162725* + CA19-9 and IGF-1 + Albumin + CA19-9 are statistically significant on the one side; on the other, this future research could determine the cost for health care systems versus the benefit of the three markers in case of significant improvement.

Circulating tumour DNA has been used in clinical settings for a long time; however, other approaches such as circulating tumour RNA have yet to demonstrate their clinical usefulness for identifying early-stage cancer. The low sensitivity and the need for large volumes of plasma may contribute to this. Because of their great sensitivity and minimum sample volume, piRNAs may represent an alternate path to an accurate diagnosis. However, validation studies are necessary before they can be used in routine clinical practice. The next step is to conduct wide-scale population-based research in both prospective and retrospective samples to support or refute the predictive capacity of the current consensus signature.

Since the piRNA/PIWI complex performs epigenetic programming, not only is it crucial to know the effect of this complex on cell biology but also its targets and the molecular pathways involved. It has been previously described that the potential *piR-017061*/PIWIL1 complex can modulate the expression of EFNA5 mRNA by direct binding, which enables its degradation, while the absence of *piR-017061* leads to EFNA5 accumulation, thereby promoting PDAC development [44]. In our study we aimed to go further, and we speculated as to which transcripts could be modulated by this potential RNA/protein complex formed with *piR-162725* and PIWIL3, and also which signalling pathway could be involved. We found several transcripts to be potentially modulated by *piR-162725*. KEGG pathway analysis revealed that most of the transcripts were significantly involved in the tumourigenic MAPK, TGF-beta, and Hippo signalling pathways. This fact suggested that *piR-162725* could be modulating a proliferative, migrative, and invasive phenotype on the one hand, and EMT, cell differentiation and metabolism on the other. To narrow our search from 317 transcripts, we evaluated their significant positive or negative correlation with PIWIL3 expression in PDAC samples from a public repository. Six downregulated transcripts in the PDAC samples suggested a potential protective effect on PDAC, four had a negative correlation with PIWIL3 expression (SHC3, RIMS3, SH3BP2, and P2RY6) and two were positively correlated with PIWIL3 (CACNA1E and TBC1D16). Furthermore, three other transcripts were upregulated in PDAC samples: SOGA1 and RAB3B showing a positive correlation with PIWIL3 expression, and P2RY2 exhibiting a negative correlation with PIWIL3. Since these three transcripts were upregulated in PDAC samples independently of their positive or negative correlation with PIWIL3 expression, they may act as tumourigenic factors. Only P2RY2 expression has been previously described in PDAC associated with poor prognosis, and the inhibition of this factor impaired tumour growth in xenograft and orthotopic PDAC models [51]. However, of the notable factors found to be upregulated in PDAC samples were SOGA1 (Suppressor of Glucose Autophagy-Associated 1) and RAB3B (Ras-Related Protein 3B). Interestingly, SOGA1 is a circRNA that suppresses *hsa-miR-21-5p* activity, thereby upregulating its related target genes [52] and RAB3B expression is modulated by circRNAs [53]. Of note, we found two oncogenes potentially regulated by the potential *piR-162725*/PIWIL3 complex, SOGA1 and RAB3B, and which could be potentially modulated through a circRNA-mRNA regulation network. We hypothesise that the potential *piR-162725*/PIWIL3 complex may also be involved in circRNA-mRNA regulation although further experiments are needed to confirm this.

## 5. Conclusions

Our findings support *piR-162725* as a potential biomarker for the identification of PDAC patients by liquid biopsy. When combined with CA19-9, *piR-162725* raises the sensitivity of CA19-9, but it still needs to be standardized for early diagnosis to improve the identification of the early stages of PDAC in patients in clinical practice. Prediction models of the potential *piR-162725*/PIWIL3 complex revealed several target transcripts that could be involved in crucial signalling pathways, displaying both a protective and oncogenic role, which could point to new strategies for the treatment of PDAC. However, further studies are needed to confirm these results and to establish the direct or indirect regulation of these transcripts by the potential *piR-162725*/PIWIL3 complex.

## Figures and Tables

**Table 1:** COX proportional hazard model for clinico-pathological characteristics of patients.

	Univariate PFS (95% CI)				Univariate OS (95% CI)			
	HR	Lower	Upper	*p*	HR	Lower	Upper	*p*
**Gender** (Female vs. Male)	1.252	0.637	2.460	0.514	1.376	0.656	2.889	0.398
**Stage** (I/II vs. III/IV)	2.467	1.137	5.353	0.022	5.545	1.909	16.109	0.002
**pT** (0–I vs. II–III)	1.417	0.641	3.131	0.389	3.302	1.134	9.616	0.029
**pN** (N0 vs. N1/N2/N3)	1.498	0.612	3.668	0.377	1.151	0.462	2.867	0.763
**pM** (M0 vs. M1)	2.062	1.047	4.061	0.036	5.840	2.386	14.298	0.001
**Alcohol** (No vs. Yes)	1.331	0.621	2.851	0.462	1.519	0.650	3.553	0.334
**Smoker** (Yes vs. No)	1.371	0.702	2.678	0.335	1.782	0.860	3.693	0.120
**Diabetes** (No vs. Yes)	1.278	0.638	2.560	0.489	1.343	0.637	2.830	0.439
**AHT** (No vs. Yes)	1.292	0.444	3.759	0.638	1.314	0.338	5.111	0.694
	**Multivariate PFS (95% CI)**				**Multivariate OS (95% CI)**			
**Stage** (I/II vs. III/IV)	1.943	0.658	5.738	0.229	10.512	0.373	296.493	0.167
**pM** (M0 vs. M1)	1.322	0.515	3.393	0.562	3.391	1.189	9.673	0.022
**pT** (0–I vs. II–III)	-	-	-	-	4.995	0.191	130.586	0.334

Abbreviations. pT: tumour size according to AJCC 8th edition. pN: number of lymph nodes affected. pM: presence of metastatic disease. AHT: arterial hypertension. HR: hazard ratio. CI: confidence interval. *p*-values < 0.05 were considered statistically significant.

## Data Availability

Results of the NGS can be found in Table S1.

## Supplementary Materials

The following supporting information can be downloaded at: https://www.mdpi.com/article/10.3390/jcm11247310/s1. Table S1 shows all the integration analyses performed with next-generation sequencing. Table S2 shows all the potential targets of *piR-162725* and their expression profile and correlation with PIWIL3 expression.

## Abbreviations

PDAC	Pancreatic ductal adenocarcinoma
PIWI	P-element-induced wimpy testis
AGO	Argonaute protein family
piRNAs	PIWI-interacting RNAs
piRISC	piRNA-induced silencing complex
TERT	Telomerase reverse transcriptase
WB	Western blot
IHC	Immunohistochemistry
siRNA	Short interference RNA
EMT	Epithelial-to-mesenchymal transition
FOLFIRINOX	5-fluorouracil, leucovorin, irinotecan, and oxaliplatin
Nab-Paclitaxel	Nano albumin-bound paclitaxel
TCGA	The Cancer Genome Atlas
FDA	Food and Drug Administration
KEGG	Kyoto Encyclopaedia of Genes and Genomes
